# Controllable Preparation of Ag-SiO_2_ Composite Nanoparticles and Their Applications in Fluorescence Enhancement

**DOI:** 10.3390/ma16010201

**Published:** 2022-12-26

**Authors:** Luping Tang, Chen Liao, Yingqing Guo, Yangyang Zhang

**Affiliations:** 1 College of Mechanical and Electrical Engineering, Nanjing Forestry University, Nanjing 210037, China; 2 College of Electronic and Optical Engineering & College of Flexible Electronics (Future Technology), Nanjing University of Posts and Telecommunications, Nanjing 210023, China; 3 SEU-FEI Nano-Pico Center, Key Lab of MEMS of Ministry of Education, Southeast University, Nanjing 210096, China

**Keywords:** Ag-SiO_2_, fluorescence enhancement, transmission electron microscope, quantum dots, photoexcitation spectra

## Abstract

Metal nanoparticles have attracted a great deal of interest due to their unique properties of surface plasmon resonance. Metal nanoparticles can enhance the fluorescence emission intensity of quantum dots (QDs) through the local surface plasmon resonance effect, which is mainly determined by the distance between them. Therefore, it is very important to achieve controllable distance between metal and QDs, and study fluorescence enhancement. In this work, the controllable adjustment of the distance between metal nanoparticles and QDs was successfully realized by controlling the thickness of the SiO_2_ shell of Ag@SiO_2_ nanoparticles. Firstly, Ag nanoparticles with uniform size distribution and relatively high concentration were prepared, and then the thickness of the SiO_2_ shell was controlled by controlling the amount of tetra-ethyl orthosilicate (TEOS) in the hydrolysis of TEOS reaction. (3-aminopropyl) triethoxysilane (APS) was used to connect CdS/ZnS QDs with Ag@SiO_2_ nanoparticles to form Ag@SiO_2_@CdS/ZnS QD composite nanoparticles. The fluorescence spectra shows that the fluorescence intensity of the Ag@SiO_2_@CdS/ZnS QD composite nanoparticles is significantly enhanced. Photoexcitation spectra and fluorescence spectra of CdS/ZnS QD and Ag@SiO_2_@CdS/ZnS QD composite nanoparticles, measured under different energy excitation conditions, indicate that the existence of Ag nanoparticles can enhance the fluorescence intensity of CdS/ZnS QDs. Finally, a further physical mechanism of fluorescence enhancement is revealed.

## 1. Introduction

Fluorescence technology plays a very important role in the modern spectral technology field, which has been widely used in many scientific and technological fields, due to its advantages such as convenient use, wide application, and high sensitivity [1,2,3]. However, in practical applications, due to the limitations of some special factors, the sensitivity of fluorescent signals still cannot meet the detection requirements, to some extent. In order to solve these problems, the fluorescence signal can be improved through various methods. For example, a fluorescent probe excited by a long wave band is used to reduce the background noise [4]; the detection sensitivity can be improved by adopting new technologies, and improving the performance of the instrument [5]. However, there are limitations in experimental states and at the technical level.

Metal nanoparticles have become one of the most popular research hotspots in recent years because of their distinctive properties of surface plasmon resonance [6,7]. Metal nanoparticles can enhance the fluorescence emission intensity of fluorescent probes through the local surface plasmon resonance effect, to improve the signal strength and signal-to-noise ratio of the detection system, based on QDs [8,9]. In the 1970s, Drexhage first reported that the rough silver electrode had a certain augmenting effect on the fluorescence emission of fluorescent materials near its surface. Later, a lot of relevant experiments and theoretical research were carried out [10]. Since 1999, Professor Lakowicz’s leading group has conducted a large number of studies on surface enhanced fluorescence and has made certain achievements. Ref. [11] Up to now, remarkable achievements have been made in the research on surface fluorescence enhancement effect, both theoretically and experimentally. Theoretically, the theory of local field enhancement, the theory of radiation attenuation rate increase, and the theory of energy transfer, based on the Mie theory, have been put forward successively [12,13]. In addition, with the development of science, the finite-difference time-domain method has begun to be used for electric field simulation and numerical calculation of specific models, which can directly show the local electric field distribution around the stimulated nanoparticles, and more clearly reflect various enhancement mechanisms [14]. In the experiment, some research achievements based on the surface Raman enhancement have been made, and a variety of effective enhancement substrates such as nano island films, ordered array nano films, nanorods, and nanoparticles have been prepared [15]. Moreover, it has been found that the nanostructures of some precious metals, such as silver, gold, and copper can achieve metal surface fluorescence enhancement [16,17,18].

Semiconductor quantum dots (QDs) have attracted great interest due to their excellent optical properties and potential biological and biomedical applications [19]. When QD probes are used in biological detection, the fluorescence signal of QDs may become very weak, or even completely annihilated by noise. The fluorescence intensity of probes is an important indicator in biological detection, so it is necessary to further improve the fluorescence intensity of QD probes in the objects to be detected [20]. If precious metal nanoparticles and QDs are combined to form a composite probe, precious metal nanoparticles will increase the fluorescence intensity of QDs, and thus solve the problem of weak signal intensity of quantum dots in biological detection. There are two main ways of metal enhanced fluorescence, one is based on metal substrate surface enhanced fluorescence, and the other is based on liquid phase system enhanced fluorescence. Refs. [21,22] In practical applications, especially in biological detection, the enhancement process needs to be realized in the liquid environment, and the fluorescence enhancement effect of metal nanoparticles on QDs is mainly determined by the distance between them [23]. Therefore, it is very important to achieve controllable distance between metal and QDs in a liquid environment and study the surface fluorescence enhancement of metal nanoparticles.

In this paper, silver nanoparticles with uniform size distribution were prepared by the chemical reduction method, and the thickness of the SiO_2_ shell was controlled by controlling the amount of tetraethyl orthosilicate (TEOS) in the hydrolysis of TEOS. Then, the water-soluble CdS/ZnS QDs with the fluorescence peak at 449 nm were connected to Ag@SiO_2_ nanoparticles to form Ag@SiO_2_@CdS/ZnS composite nanoparticles. By measuring the fluorescence spectra, it was found that the fluorescence of the Ag@SiO_2_@CdS/ZnS composite nanoparticles is 3.5 times stronger than that of CdS/ZnS QDs. Finally, the mechanism of metal enhanced fluorescence has been investigated. 

## 2. Materials and Methods

### 2.1. Materials

Silver nitrate (AgNO_3_, 99%), polyvinylpyrrolidone (PVP, 99%), tetraethyl orthosilicate (TEOS, 98%), and 3-Mercaptopropionic acid (MPA, 98%) were purchased from Aladdin Reagent Company (Shanghai, China). Ethylene glycol, acetone, ethanol, ammonia (NH_3_·H_2_O, 20–30%), and (3-aminopropyl) triethoxysilane (APS) were purchased from Sinopharm Chemical Reagent Co., Ltd. (Shanghai, China). All reagents were used directly without further purification. In addition, deionized water was used as the solvent for all solution preparations in the reaction process.

### 2.2. Preparation of Ag Nanoparticles

An amount of 0.5 g PVP was dissolved in 15 mL ethylene glycol and heated slowly to 180 °C, under stirring. The temperature was adjusted to 120 °C for half an hour. A total of 0.1 g AgNO_3_ was dissolved in 5 mL ethylene glycol and then injected into the above mixed solution, drop by drop. After reacting for two minutes, the heating was stopped, and the solution cooled to room temperature. Excessive acetone was added to produce a brown precipitate, which was finally dissolved in 8 mL deionized water and ultrasonicated for 30 min.

### 2.3. Preparation of Ag@SiO_2_ Nanoparticles

A total of 2 mL of the prepared silver nanoparticles were added into conical flasks (No. 1–7) filled with 20 mL ethanol, under stirring conditions. A volume of 200 μL (28–30%) of NH_3_·H_2_O was added, and then 5, 10, 15, 20, 25, 40, and 80 μL TEOS were added into the conical flasks (No. 1–7), under stirring at 30 °C for 8 h, respectively. Finally, the solution was purified through centrifuging under 9562× *g* for 10 min, and the precipitates were redissolved in 4 mL ethanol and stored at 4 °C.

### 2.4. Preparation of Ag@SiO_2_@CdS/ZnS Composite Nanoparticles

A volume of 2 mL of the above prepared Ag@SiO_2_ nanoparticles (No. 2, 10 μL TEOS) and 20 μL APS were mixed, under stirring conditions, at room temperature, for 8 h. The reaction solution was centrifuged at 15,000 rpm for 20 min, followed by the removal of the supernatant, and then the sediment was dissolved in a certain volume of ethanol. The centrifugation separation process was repeated 3–4 times to ensure that the excess APS was removed completely, and finally the sediment was dissolved in 2 mL ethanol. A total of 80 μL water-soluble CdS/ZnS QDs solution was added into the purified Ag@SiO_2_ solution, under stirring, for 1–2 h, at room temperature. After the reaction, the sample was centrifugated at 15,000 rpm for 15 min, and then the sediment was dissolved in ethanol. The above process was repeat 3 times, and then the sample was dissolved in 2 mL ethanol, and stored at 4 °C for measurement.

### 2.5. Characterization Method

The absorption spectra of the samples were measured by Shimadzu UV-3600 PC spectrophotometer. TEM images were measured by FEI Tecnai G2 (200 kV). The fluorescence spectra, fluorescence lifetime spectra, and the photoexcitation spectra were obtained by fluorescence spectrometer (F900, Edinburgh instrument).

## 3. Results and Discussion

**Figure 1:**
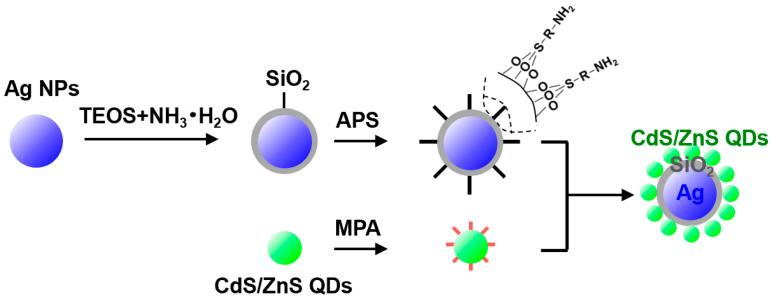
Schematic diagram of preparing Ag@SiO_2_@CdS/ZnS QD composite nanoparticles.

In the liquid phase system, the distance between metal nanoparticles and QDs is one of the most important factors affecting metal-enhanced fluorescence [24,25]. The controllable adjustment of the distance between metal nanoparticles and QDs was successfully observed by controlling the thickness of the SiO_2_ shell of Ag@SiO_2_ nanoparticles. Figure 1 shows the schematic diagram of the preparation of Ag@SiO_2_@CdS/ZnS QD composite nanoparticles. Firstly, Ag nanoparticles with uniform size distribution and relatively high concentration were prepared, and then the thickness of the silica shell was controlled by controlling the amount of tetraethyl orthosilicate (TEOS) in the hydrolysis of TEOS reaction. (3-aminopropyl) triethoxysilane (APS, a silane coupling agent) was used to connect CdS/ZnS QDs with Ag@SiO_2_ nanoparticles to form Ag@SiO_2_@CdS/ZnS QD composite nanoparticles. Specifically, the Ag/SiO_2_ core/shell nanoparticle (NP) was treated with APS, which reacted with surface silanol groups to generate a silicon dioxide surface, with NH_2_ group functionalization. Then, water-soluble CdS/ZnS QDs, which were synthesized based our previous protocol [26], were added to the ethanol solution of the aminated Ag@SiO_2_ core-shell nanoparticles and stirred for 1.5 h at room temperature. Finally, the optical properties of Ag@SiO_2_@CdS/ZnS QD composite nanoparticles were measured.

**Figure 2:**
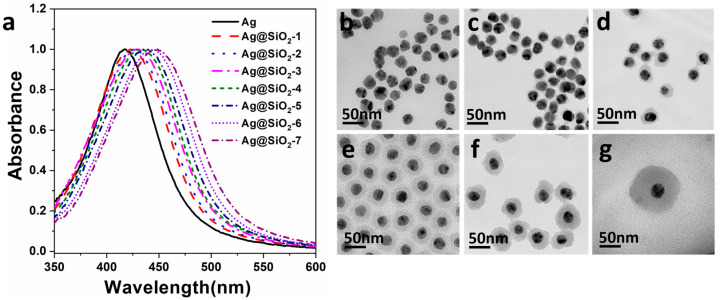
(**a**) The absorption spectra of silver (Ag) nanoparticles (black solid line) and the absorption spectra of Ag@SiO_2_ samples prepared by adding 5, 10, 15, 20, 25, 40, and 80 μL TEOS (dashed line from left to right); TEM images of Ag nanoparticles (**b**) and Ag@SiO_2_ samples with SiO_2_ thickness of (**c**) 2 nm, (**d**) 12 nm, (**e**) 18 nm, (**f**) 20 nm, and (**g**) 35 nm, respectively.

Figure 2a shows the absorption spectra of Ag nanoparticles and Ag@SiO_2_ nanoparticles with different thicknesses of SiO_2_ shells. It can be seen that the absorption peak of Ag nanoparticles is 417 nm. Volumes of 5, 10, 15, 20, 25, 40, and 80 µL TEOS were added to prepare Ag@SiO_2_ with different thicknesses, respectively. The absorption spectra of Ag@SiO_2_ nanoparticles showed different degrees of red shift, and their corresponding absorption peaks were 422, 425, 427, 432, 437, 443, and 448 nm, respectively. The occurrence of the red shift is directly related to the change of the refractive index of the surrounding medium and the surface properties of silver nanoparticles by the coating of SiO_2_ shells with different thicknesses [27].

In order to verify the thicknesses of the SiO_2_ shell on Ag nanoparticles, it was necessary to further measure the TEM images of these series of samples. Figure 2b shows the transmission electron microscope (TEM) image of Ag nanoparticles with an average diameter of 30 nm. Figure 2c–g displays the TEM images of Ag@SiO_2_ prepared with 5, 10, 20, 40, and 80 μL TEOS, respectively. SiO_2_ shell thicknesses, as seen in Figure 2c–g, were measured using the TEM images, which measured 2, 12, 18, 20, and 35 nm, respectively. Obviously, the samples have core-shell structure with good size homogeneity, in which the darker part in the middle is composed of Ag nanoparticles and the lighter surrounding area is the coated SiO_2_ shell. Moreover, it can be deduced from the TEM images that, within a certain range, the more TEOS added, the thicker the SiO_2_ shell coat is.

**Figure 3:**
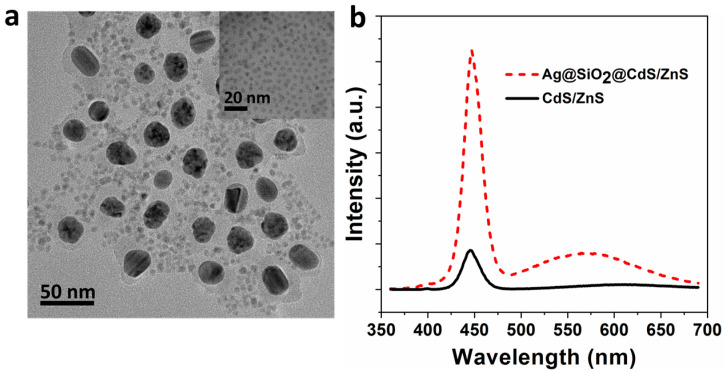
(**a**) TEM images of Ag@SiO_2_(12 nm)@CdS/ZnS QD composite nanoparticles. Inset is the TEM image of CdS/ZnS QDs; (**b**) Fluorescence spectra of CdS/ZnS QDs (black solid line) and Ag@SiO_2_(12 nm)@CdS/ZnS QD composite nanoparticles (red short dash line), respectively. The excitation wavelength of the fluorescence spectrum is 377 nm.

**Figure 4:**
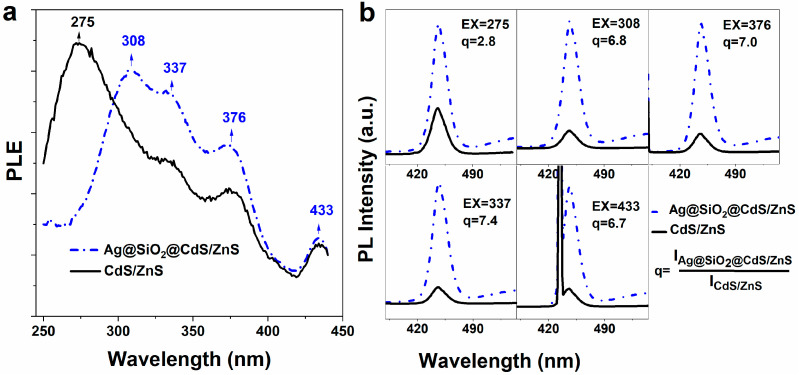
(**a**) Photoexcitation spectra of CdS/ZnS QD (black solid line) and Ag@SiO_2_(12 nm)@CdS/ZnS QD composite nanoparticles (blue short dash dot line), respectively; (**b**) Fluorescence spectra of CdS/ZnS QD (black solid line) and Ag@SiO_2_(12 nm)@CdS/ZnS QD composite nanoparticles measured under different energy excitation conditions, respectively, where EX represents the excitation light wavelength and q represents the ratio of the intrinsic fluorescence peak area (with an integrated wavelength range of 420–480 nm) of CdS/ZnS QD (black solid line) and Ag@SiO_2_(12 nm)@CdS/ZnS QD composite nanoparticles (blue short dash dot line).

Figure 3a displays the TEM image of Ag@SiO_2_(12 nm)@CdS/ZnS QD composite nanoparticles. To evaluate the fluorescence intensity enhancement of the Ag@SiO_2_(12 nm)@CdS/ZnS QD composite nanoparticles, compared to CdS/ZnS QDs, the absorption intensities of the two samples at the excitation wavelength (377 nm) were adjusted to be consistent by changing the concentration. The fluorescence spectra of Ag@SiO_2_(12 nm)@CdS/ZnS QD composite nanoparticles and CdS/ZnS QDs are represented in Figure 3b. The intrinsic and defect fluorescence of Ag@SiO_2_@CdS/ZnS QD composite nanoparticles are located at 449 nm and 571 nm, respectively. Obviously, the intrinsic fluorescence intensity (5 times) and the defect fluorescence intensity (12 times) of Ag@SiO_2_(12 nm)@CdS/ZnS QD composite nanoparticles were found to be enhanced. In addition, we also measured the absolute fluorescence quantum yield. The quantum yields of Ag@SiO_2_(12 nm)@CdS/ZnS QD composite nanoparticles and CdS/ZnS QDs are about 80% and 9%, respectively. In order to explore the experimental mechanism of this phenomenon, photoexcitation spectra of CdS/ZnS QD (black solid line) and Ag@SiO_2_(12 nm)@CdS/ZnS QD composite nanoparticles (blue short dash dot line) were measured, respectively, as shown in Figure 4a. The emission wavelength of the two fluorescence intensities is 449 nm, which is the location of the intrinsic peak. The measurement principle of photoexcitation spectrum is to fix a certain wavelength as the detection wavelength (generally the peak position of the sample luminous peak) by constantly changing the excitation energy (wavelength), and recording the fluorescence intensity at the detection wavelength [28]. The curve composed of these intensity values shows the contribution of excitation light of different energies to the photoluminescence of the detected wavelength. Photoexcitation spectroscopy can provide information about the structure, composition, and atomic arrangement of materials [29]. It is a non-destructive and highly sensitive analysis method. As can be seen from Figure 4a, Ag@SiO_2_(12 nm)@CdS/ZnS QD composite nanoparticles show a different photoexcitation spectrum from CdS/ZnS QDs, due to the influence of Ag nanoparticles. Compared with CdS/ZnS QDs, the signal peak intensity is relatively close at 433 nm, while there is no signal peak at 275 nm in the photoexcitation spectrum of Ag@SiO_2_(12 nm)@CdS/ZnS QD composite nanoparticles, and the signal intensity is significantly enhanced at 308 nm, 337 nm, and 376 nm.

To further analyze the experimental results, the fluorescence spectra of CdS/ZnS QD and Ag@SiO_2_(12 nm)@CdS/ZnS QD composite nanoparticles measured under different energy excitation conditions were acquired. Figure 4b displays the fluorescence spectra of CdS/ZnS QD (black solid line) and Ag@SiO_2_(12 nm)@CdS/ZnS QD composite nanoparticles (blue short dash dot line) under excitation light wavelengths of 275, 308, 337, 376, and 433 nm. Interestingly, when the excitation wavelengths are 275 nm, 308 nm, 337 nm, 376 nm, and 433 nm, the ratio of the intrinsic fluorescence intensity of Ag@SiO_2_(12 nm)@CdS/ZnS QD composite nanoparticles and CdS/ZnS QDs is 2.8, 6.8, 7.4, 7.0, and 6.7, respectively, showing a trend, which increases first, before decreasing. This is consistent with the result given by the photoexcitation spectra. Compared with CdS/ZnS QDs, the excitation light with the wavelength of 275 nm cannot effectively excite the fluorescence of Ag@SiO_2_(12 nm)@CdS/ZnS QD composite nanoparticles, while the excitation light with the wavelengths of 308, 337, and 376 nm can excite the fluorescence of Ag@SiO_2_(12 nm)@CdS/ZnS QD composite nanoparticles more. When the excitation wavelength is 433 nm, the ratio of the intrinsic fluorescence intensity of Ag@SiO_2_(12 nm)@CdS/ZnS QD composite nanoparticles to CdS/ZnS QDs decreases. All these phenomena indicate that the existence of Ag nanoparticles can enhance the fluorescence intensity of CdS/ZnS QDs.

**Figure 5:**
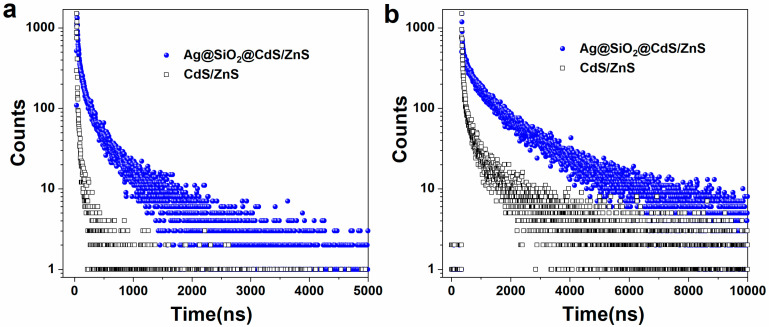
The time-resolved intrinsic fluorescence intensity (**a**) and defect fluorescence intensity (**b**) of CdS/ZnS QD (black open square) and Ag@SiO_2_ (12 nm)@CdS/ZnS QD composite nanoparticles (blue sphere).

Figure 5 shows the time-resolved intrinsic fluorescence intensity (449 nm) and defect fluorescence intensity (571 nm) of CdS/ZnS QD and Ag@SiO_2_(12 nm)@CdS/ZnS QD composite nanoparticles. The intrinsic state and defect state lifetimes of CdS/ZnS QDs determined by double exponential fitting are 128 ns and 977 ns, respectively. The intrinsic state and defect state lifetimes of Ag@SiO_2_(12 nm)@CdS/ZnS QD composite nanoparticles are 332 ns and 1643 ns, respectively. It can be seen from Figure 3 and Figure 5 that the fluorescence intensity and fluorescence lifetime of Ag@SiO_2_(12 nm)@CdS/ZnS QD composite nanoparticles are enhanced, compared to CdS/ZnS QDs. This experimental phenomenon is different from previous research reports [30]. Most of the phenomena observed in previous studies are fluorescence enhancement, and the lifetime is shortened. However, the phenomena in this experiment did not happen by accident. Similar experimental phenomena have also been observed in the research by the Lian Hu research group [31,32] and Tuncay Ozel research group [33]. When the spacing between metal nanoparticles and QDs is appropriate, the SiO_2_ shell can effectively inhibit the energy transfer from the QDs to the metal nanoparticles. In addition, the interaction between the metal nanoparticles and QDs may weaken the original non-radiation path of the QDs. The change of the non-radiation decay rate is defined as $k_{0}$ ($k_{0}>0$), and then the non-radiation decay rate of the QDs, $k_{nr}$, becomes $k_{nr}-k_{0}$. At this time, the fluorescence quantum yield (Q) and fluorescence lifetime (T) of CdS/ZnS QDs can be expressed as [30]:

(1) $$Q=k_{r}/\left(k_{r}+k_{nr}-k_{0}\;\right)$$

(2) $$T={\left(k_{r}+k_{nr}-k_{0}\right)}^{-1}$$

It can be seen from Formulas (1) and (2) that the non-radiative decay rate decreases, the quantum yield increases, and the fluorescence lifetime lengthens, which is consistent with our experimental phenomenon.

## 4. Conclusions

In summary, Ag@SiO_2_ nanoparticles were synthesized by the colloidal method, and the thickness of SiO_2_ shell was controlled by controlling the amount of TEOS. The controllable adjustment of the distance between metal nanoparticles and QDs was successfully realized by controlling the thickness of the SiO_2_ shell of Ag@SiO_2_ nanoparticles. Then, CdS/ZnS QDs were connected with Ag/SiO_2_ nanoparticles to investigate the fluorescence enhancement. Through the analysis of the experimental results, it was found that the fluorescence intensity was enhanced significantly, and that the fluorescence lifetime was also extended. Compared with CdS/ZnS QDs, the intrinsic fluorescence intensity of the Ag@SiO_2_@CdS/ZnS QD composite nanoparticles increased by 5 times and the defect fluorescence intensity increased by 12 times. The results further revealed the physical mechanism of fluorescence enhancement of Ag@SiO_2_@CdS/ZnS QD composite nanoparticles, in which the SiO_2_ shell can effectively inhibit the energy transfer from the QDs to the metal nanoparticles. As a result, the interaction between the metal nanoparticles and QDs may weaken the original non-radiation path of the QDs, when the spacing between metal nanoparticles and QDs is appropriate.

## Data Availability

Not applicable.
